# Inhibition of Staphylococcus aureus Biofilm Formation and Virulence Factor Production by Petroselinic Acid and Other Unsaturated C18 Fatty Acids

**DOI:** 10.1128/spectrum.01330-22

**Published:** 2022-06-01

**Authors:** Jin-Hyung Lee, Yong-Guy Kim, Jintae Lee

**Affiliations:** a School of Chemical Engineering, Yeungnam University, Gyeongsan, Republic of Korea; Penn State University

**Keywords:** biofilm, hemolysis, fatty acids, petroselinic acid, *Staphylococcus aureus*

## Abstract

Staphylococcus aureus is a major human pathogen that secretes several toxins associated with the pathogenesis of sepsis and pneumonia. Its antibiotic resistance is notorious, and its biofilms play a critical role in antibiotic tolerance. We hypothesized fatty acids might inhibit S. aureus biofilm formation and the expressions of its virulence factors. Initially, the antibiofilm activities of 27 fatty acids against a methicillin-sensitive S. aureus strain were investigated. Of the fatty acids tested, three C18 unsaturated fatty acids, that is, petroselinic, vaccenic, and oleic acids at 100 μg/mL, inhibited S. aureus biofilm formation by more than 65% without affecting its planktonic cell growth (MICs were all > 400 μg/mL). Notably, petroselinic acid significantly inhibited biofilm formation of two methicillin-resistant S. aureus strains and two methicillin-sensitive S. aureus strains. In addition, petroselinic acid significantly suppressed the production of three virulence factors, namely, staphyloxanthin, lipase, and α-hemolysin. Transcriptional analysis showed that petroselinic acid repressed the gene expressions of quorum sensing regulator *agrA*, effector of quorum sensing *RNAIII*, α-hemolysin *hla*, nucleases *nuc1* and *nuc2*, and the virulence regulator *saeR*. Furthermore, petroselinic acid dose-dependently inhibited S. aureus biofilm formation on abiotic surfaces and porcine skin. These findings suggest that fatty acids, particularly petroselinic acid, are potentially useful for controlling biofilm formation by S. aureus.

**IMPORTANCE** Fatty acids with a long carbon chain have recently attracted attention because of their antibiofilm activities against microbes. Here, we report the antibiofilm activities of 27 fatty acids against S. aureus. Of the fatty acids tested, three C18 unsaturated fatty acids (petroselinic, vaccenic, and oleic acids) significantly inhibited biofilm formation by S. aureus. Furthermore, petroselinic acid inhibited the production of several virulence factors in S. aureus. The study also reveals that the action mechanism of petroselinic acid involves repression of quorum-sensing-related and virulence regulator genes. These findings show that natural and nontoxic petroselinic acid has potential use as a treatment for S. aureus infections, including infections by methicillin-resistant S. aureus strains, and in food processing facilities.

## INTRODUCTION

Staphylococcus aureus infections are common in community and hospital settings, and the bacterium produces various virulence factors that cause diverse life-threatening infections in mammalian hosts (1). Furthermore, the development of multidrug-resistant strains, such as methicillin-resistant S. aureus (MRSA) and vancomycin-resistant S. aureus, poses a serious threat to human health. Moreover, S. aureus biofilms play a central role in antibiotic tolerance (2) and the determination of disease severity and postoperative course (3).

S. aureus readily forms biofilms on many biotic and abiotic surfaces, including host cells and medical implants. Various factors such as environmental cues, quorum sensing, c-di-GMP, protease, DNase, and hemolysins contribute to biofilm formation by S. aureus (4), and thus the identification of antibiofilm and antivirulence agents that are capable of inhibiting the production of biofilms and virulence factors without allowing the bacterium to develop drug resistance has been widely studied (5, 6); more importantly, *in vivo* studies should be linked to *in vitro* studies. In this study, we sought to find novel, nontoxic agents that inhibit biofilm formation and the virulence characteristics of S. aureus without killing the bacterium.

The antibacterial activities of high doses of fatty acids have been widely investigated (7, 8), and several recent studies have reported that a few fatty acids at subinhibitory concentrations exhibit antibiofilm and antivirulence activities (9). For example, eicosenoic, eicosadienoic, linoleic/linolenic, myristoleic, and oleic acids exhibited antibiofilm activity against S. aureus biofilms (10–14). However, no study has been attempted to analyze the antibiofilm characteristics of large numbers of fatty acids against S. aureus.

In this study, 27 fatty acids were initially screened for antibiofilm activity against a methicillin-sensitive S. aureus strain (MSSA), and the most potent, petroselinic acid, was further investigated using one other MSSA and two MRSA strains. In addition, scanning electron microscopy (SEM), confocal laser scanning microscopy (CLSM), quantitative real-time reverse transcription PCR (qRT-PCR), and staphyloxanthin production, lipase activity, and hemolysis assays were utilized to investigate how petroselinic acid influences biofilm formation by and the virulence of S. aureus. We also used a porcine skin model to confirm the antibiofilm effects of petroselinic acid on S. aureus.

## RESULTS

### Antibiofilm activities of fatty acids against S. aureus.

Twenty-seven fatty acids (15 saturated and 12 unsaturated fatty acids) were initially screened at a concentration of 100 μg/mL to investigate their antibiofilm activities against S. aureus. Several inhibited biofilm formation by S. aureus MSSA 6538 but with widely different efficacies. Detailed information on biofilm inhibition by the fatty acids and their MICs is provided in Table 1. Most of the fatty acids had MICs of >400 μg/mL, though undecanoic and myristoleic acids had MICs of 200 and 100 μg/mL, respectively, after incubation for 24 h at 37°C.

**TABLE 1:** Inhibitory effects of various fatty acids on S. aureus biofilm formation and cell growth^*a*^

No.	Fatty acids (100 μg/mL)	Biofilm (%)	Cell growth (%)	MIC (μg/mL)
	None	100 ± 5.6	100 ± 5.2	
1	Butanoic acid (4:0)	101 ± 5.3	103 ± 3.8	>400
2	Pentanoic acid (5:0)	108 ± 5.9	102 ± 4.9	>400
3	Hexanoic acid (6:0)	109 ± 5.9	116 ± 8.9	>400
4	Heptanoic acid (7:0)	109 ± 8.5	132 ± 6.2	>400
5	Octanoic acid (8:0)	115 ± 4.2	145 ± 10	>400
6	Nonanoic acid (9:0)	115 ± 8.1	132 ± 9.1	>400
7	Decanoic acid (10:0)	66 ± 5.9	85 ± 1.7	>400
8	Undecanoic acid (11:0)	45 ± 11	30 ± 2.1	200
9	Lauric acid (12:0)	70 ± 25	84 ± 18	400
10	Myristic acid (14:0)	87 ± 8.1	114 ± 9.5	>400
11	Myristoleic acid (14:1 ω-5)	14 ± 4.4	7.6 ± 4.0	100
12	Palmitic acid (16:0)	70 ± 6.6	115 ± 13	>400
13	Palmitoleic acid (16:1 ω-7)	93 ± 16	90 ± 6.8	400
14	Heptadecanoic acid (17:0)	79 ± 3.9	94 ± 3.7	>400
15	Stearic acid (18:0)	104 ± 6.8	95 ± 2.7	>400
16	Vaccenic acid (18:1 ω-7)	33 ± 4.1	130 ± 9.5	>400
17	Oleic acid (18:1 ω-9, *cis*)	27 ± 1.6	147 ± 20	>400
18	Elaidic acid (18:1 ω-9, *trans*)	103 ± 9.6	97 ± 7.5	>400
19	Petroselinic acid (18:1 ω-12)	21 ± 2.5	125 ± 15	>400
20	Conjugated linoleic acid (18:2 ω-6)	41 ± 6.3	87 ± 5.3	>400
21	α-Linolenic acid (18:3 ω-3)	101 ± 3.2	152 ± 29	>400
22	γ-Linolenic acid (18:3 ω-6)	77 ± 23	145 ± 26	>400
23	Arachidonic acid (20:4 ω-6)	52 ± 4.6	134 ± 16	>400
24	Behenic acid (22:0)	92 ± 6.7	100 ± 2.9	>400
25	Erucic acid (22:1 ω-9)	80 ± 8.3	144 ± 5.6	>400
26	Tricosanoic acid (23:0)	72 ± 7.4	124 ± 9.3	>400
27	Nervonic acid (24:1 ω-9)	53 ± 9.5	96 ± 3.6	>400

a Biofilm formation and planktonic cell growth by S. aureus MSSA 6538 were measured in 96-well polystyrene plates without agitation in the presence of each fatty acid at 100μg/mL after incubation for 24 h. MICs of each fatty acid was measured in 96-well plates after incubation for 24 h at 37°C.

Notably, six fatty acids, namely, undecanoic acid (11:0), myristoleic acid (14:1), vaccenic acid (18:1 ω-7), oleic acid (18:1 ω-9, *cis*), petroselinic acid (18:1 ω-12), and conjugated linoleic acid (18:2 ω-6), at a concentration of 100 μg/mL inhibited S. aureus biofilm formation by >50%, which concurs with reports that linoleic acid (12), oleic acid (10, 15), and myristoleic acid (13) inhibit biofilm formation by S. aureus. Interestingly, unlike two fatty acids, undecanoic acid (11:0) and myristoleic acid (14:1), four C18 fatty acids, vaccenic acid (18:1 ω-7), oleic acid (18:1 ω-9, *cis*), petroselinic acid (18:1 ω-12), and conjugated linoleic acid (18:2 ω-6), at 100 μg/mL did not inhibit planktonic cell growth. Here, we report for the first time that petroselinic acid (18:1 ω-12) potently reduces biofilm formation without adversely affecting planktonic cell growth.

### Antibiofilm activity of petroselinic acid against MSSA and MRSA.

**FIG 1:**
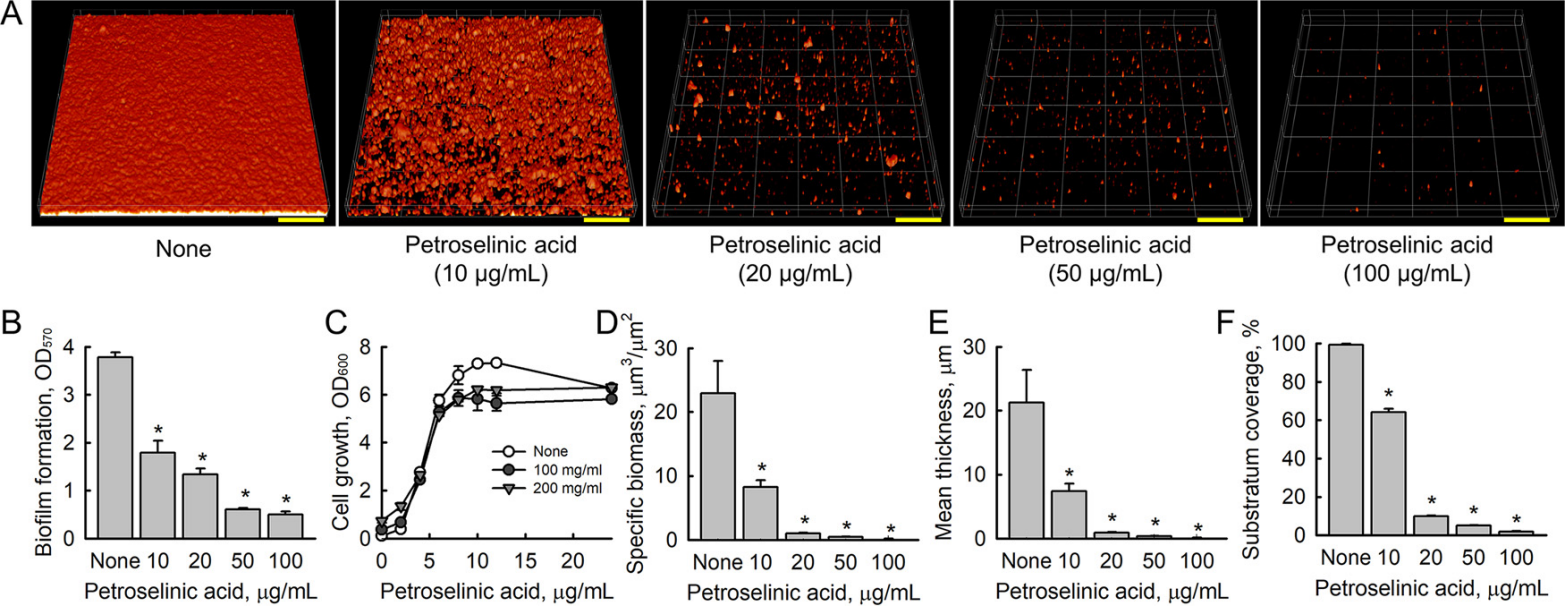
Antibiofilm activity of petroselinic acid against S. aureus. Biofilm formation by S. aureus MSSA 6538 in the presence of petroselinic acid (A, CLSM; B, Crystal violet assay). Planktonic cell growth in the presence of petroselinic acid (C) was assessed for 24 h and COMSTAT analysis was performed based on CLSM results (D–F). Scale bars represent 100 μm.

Crystal violet biofilm assays showed petroselinic acid dose-dependently inhibited MSSA 6538 biofilm formation in polystyrene plates (Fig. 1B), and petroselinic acid at concentrations up to 200 μg/mL did not inhibit the planktonic cell growth of S. aureus (Fig. 1C). Biofilm inhibition was further analyzed by CLSM. While S. aureus MSSA 6538 formed dense biofilms (thickness >20 μm and almost total surface coverage) in nontreated controls, petroselinic acid at 20 μg/mL dramatically reduced biofilm densities and thicknesses (Fig. 1A). Notably, the MIC of petroselinic acid (>400 μg/mL) was 20 times higher than the concentration required for antibiofilm activity (20 μg/mL). Biofilm reduction by petroselinic acid was confirmed by COMSTAT analysis, which showed that at 20 μg/mL, it remarkably reduced biofilm biomass, average thickness, and substrate coverage by 95, 96, and 90% as compared with untreated controls (Fig. 1D to F).

**FIG 2:**
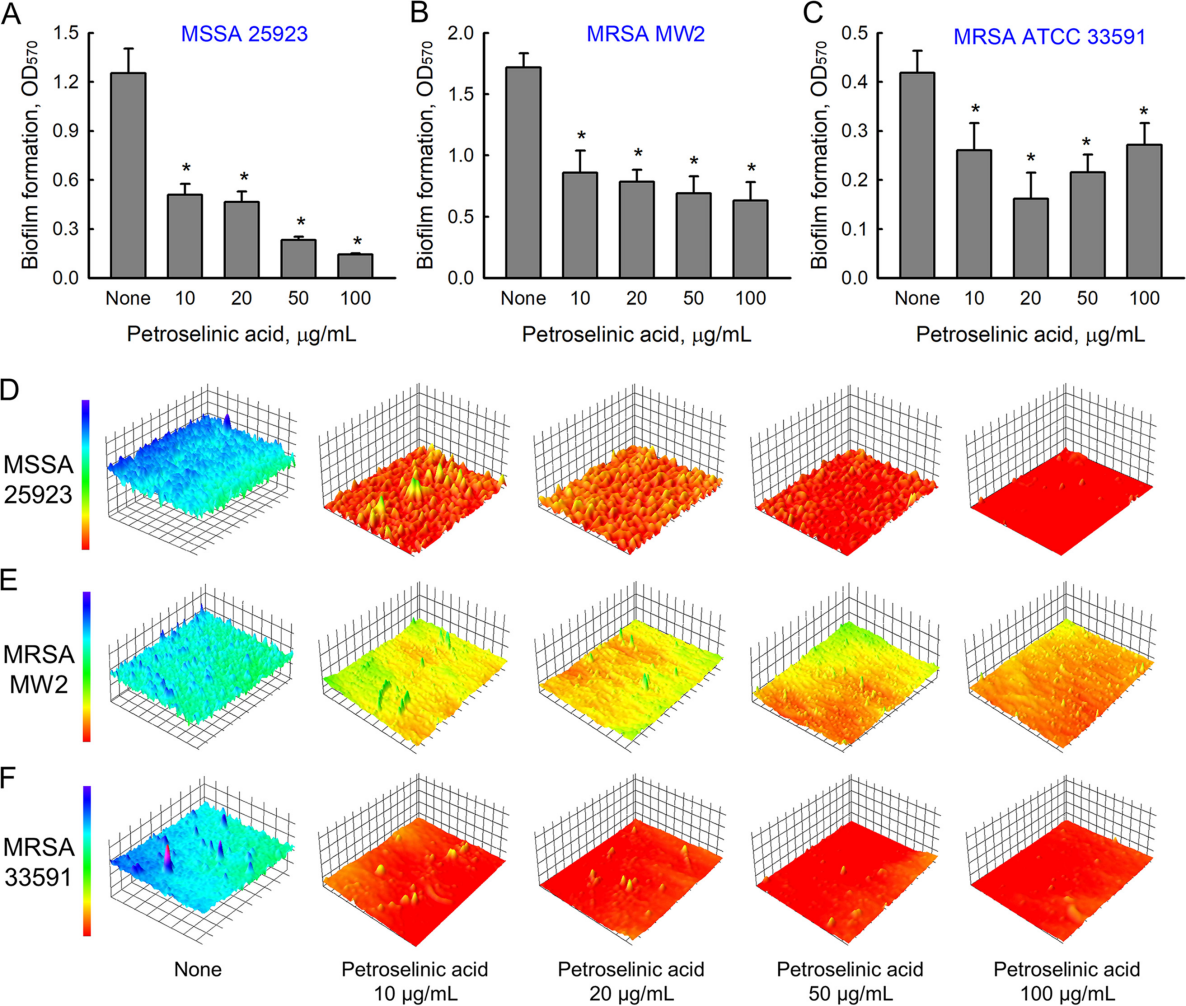
Antibiofilm activity of petroselinic acid against three other S. aureus strains, MSSA 25923 (A), MRSA MW2 (B), and MRSA 33591 (C). Color-coded 3-D images of S. aureus biofilms were generated in the presence of petroselinic acid (D–F).

**FIG 3:**
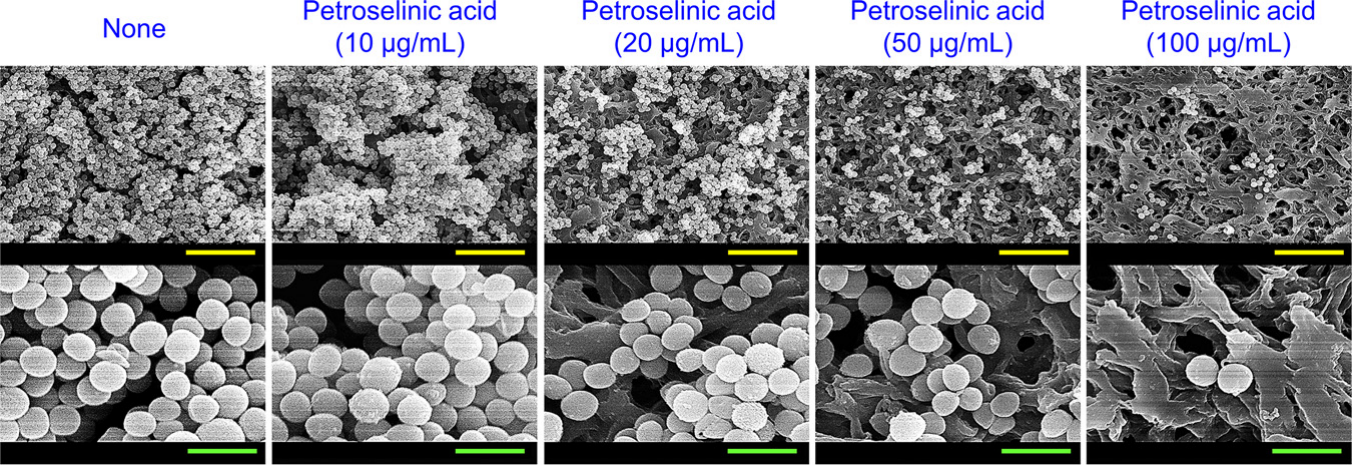
Inhibition of S. aureus biofilm formation by petroselinic acid on an abiotic surface. SEM images of S. aureus MSSA 6538 biofilms formed in the presence or absence of petroselinic acid on nylon membranes. Yellow and green scale bars represent 10 and 1.5 μm, respectively.

Biofilm assays were also performed on three other S. aureus strains (MSSA 25923, MRSA MW2, and MRSA 33591). In the absence of petroselinic acid, MSSA 25923 and MRSA MW2 formed strong biofilms, while MRSA 33591 was less effective (Fig. 2A to C). Interestingly, petroselinic acid dose-dependently inhibited biofilm development by all three S. aureus strains with MICs of >400 μg/mL, and more specifically, at 20 μg/mL decreased biofilm formation by all three S. aureus strains by ≥50%. Bright-field microscopy using 2-D and 3-D LUT mesh plots showed that petroselinic acid at 10–100 μg/mL dose-dependently reduced biofilm formation on the bottom of polystyrene plates (Fig. 2D to F), and SEM confirmed that petroselinic acid dose-dependently decreased S. aureus cell densities in biofilms (Fig. 3). However, at concentrations up to 100 μg/mL, petroselinic acid had minimal effects on cell morphology, indicating it had no antibacterial activity at these concentrations.

### Petroselinic acid suppressed the production of virulence factors.

**FIG 4:**
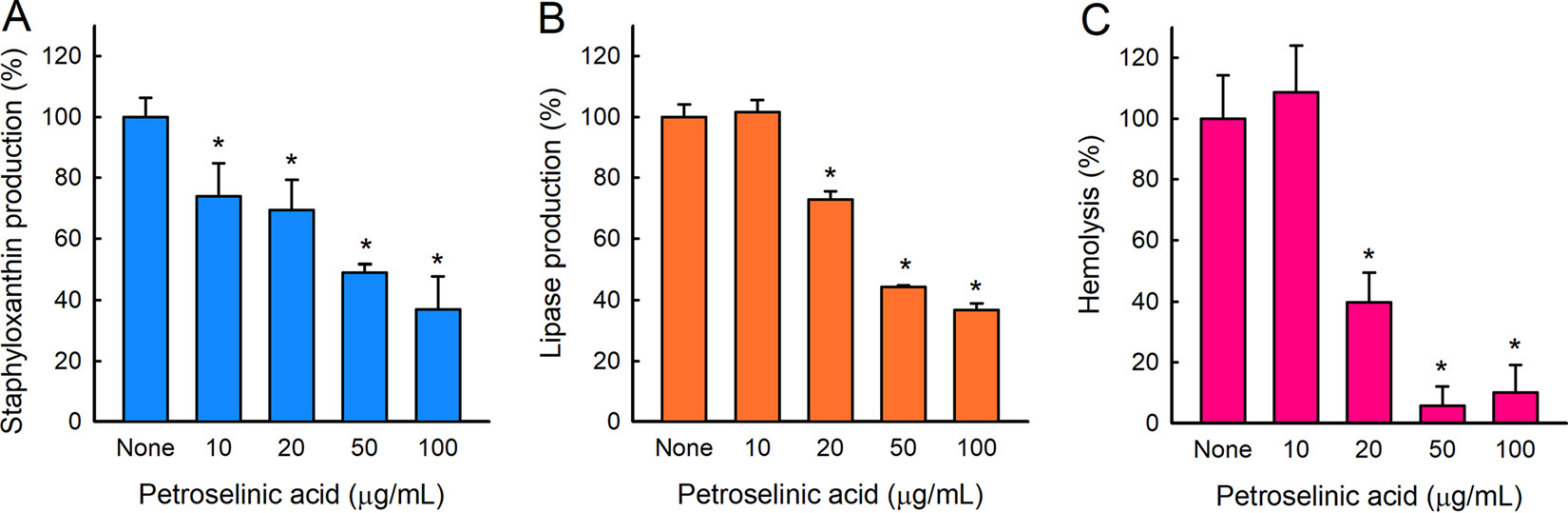
Effect of petroselinic acid on the production of staphyloxanthin (A), lipase (B), and hemolysin (C) in S. aureus MSSA 6538.

Since S. aureus produces several virulence factors, such as staphyloxanthin, hemolysins, and lipase, we investigated the effects of petroselinic acid on their production. Petroselinic acid dose-dependently reduced staphyloxanthin production without affecting cell morphology and growth (Fig. 4A). For example, petroselinic acid at 50 μg/mL inhibited staphyloxanthin production by more than 50%, and dose-dependently inhibited lipase production (e.g., by more than 60% at 50 μg/mL) (Fig. 4B) and human red blood cell hemolytic activity (e.g., by more than 90% at 50 μg/mL) (Fig. 4C).

### Transcriptional changes induced by petroselinic acid in S. aureus.

**FIG 5:**
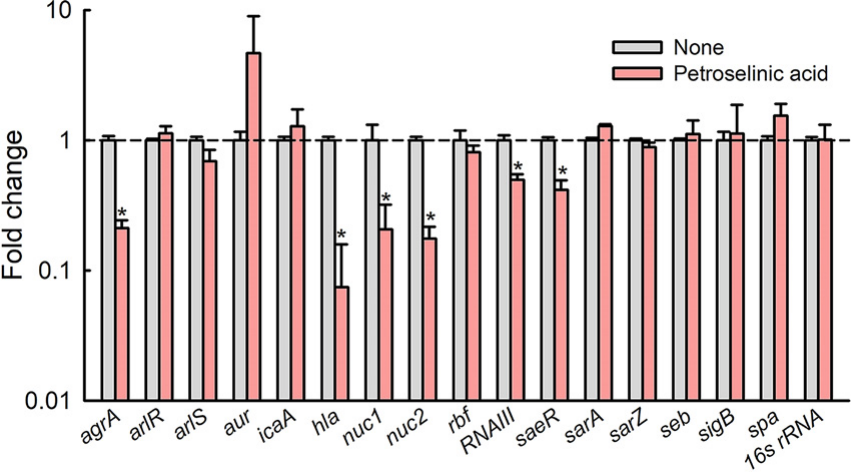
Relative transcriptional profiles of S. aureus cells treated with petroselinic acid at 100 μg/mL for 6 h. Transcriptional profiles were acquired by qRT-PCR. Fold changes delineate changes in the gene transcriptions of treated *versus* non-treated S. aureus MSSA 6538 as determined by qRT-PCR. *P < *0.05 versus nontreated cells (None).

To understand the mechanisms underlying the antibiofilm and antivirulence effects of petroselinic acid against S. aureus, we investigated its effects on the expressions of selected biofilm, virulence, and regulatory genes in S. aureus MSSA 6538 cells by qRT-PCR. Petroselinic acid downregulated the expression of quorum sensing regulator *agrA*, quorum sensing regulatory *RNAIII*, α-hemolysin *hla*, nuclease *nuc1* and *nuc2*, and the virulence regulator *saeR*, whereas the other genes tested were relatively unaffected (Fig. 5). Notably, petroselinic acid downregulated the expressions of *agrA* and *hla* by 4.7- and 13-fold, respectively.

### Petroselinic acid inhibited S. aureus adhesion to porcine skin.

**FIG 6:**
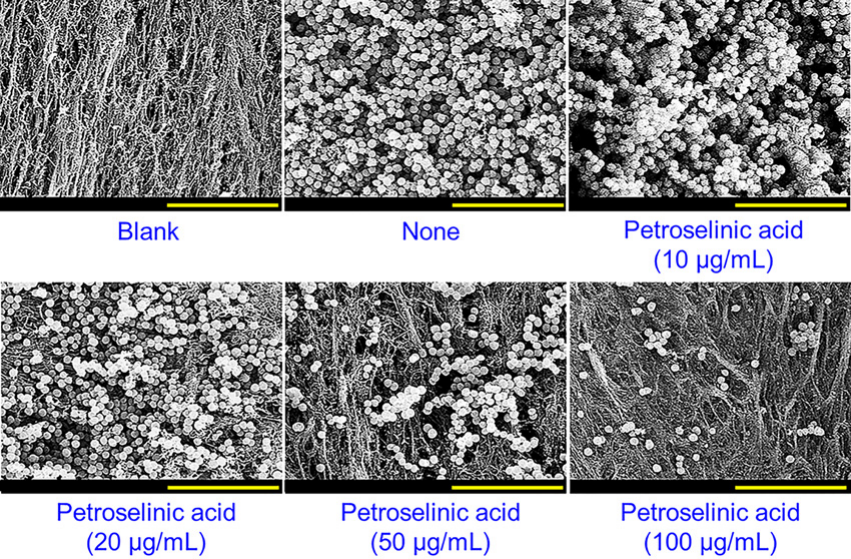
Antibiofilm effect of petroselinic acid on porcine skin. SEM images of S. aureus MSSA 6538 biofilms formed on porcine skin over 24 h. The blank shows the result obtained without bacterial treatment, and None indicates no treatment with fatty acid. Scale bars represent 10 μm.

Since S. aureus is an inhabitant of animal skin, we used SEM to assess the ability of petroselinic acid to prevent S. aureus adhesion to porcine skin (Fig. 6). Nontreated S. aureus MSSA 6538 control formed dense biofilm on porcine skin, whereas petroselinic dose-dependently inhibited cell adhesion and biofilm development. Notably, at a sub-MIC of 100 μg/mL, petroselinic acid almost prevented cell attachment.

## DISCUSSION

Fatty acids are ubiquitously present in nature, and recently, their antibiofilm and antivirulence activities have been considered as possible alternatives to conventional antimicrobials (9). Here, we report the biofilm inhibitory abilities of a series of fatty acids against S. aureus (Table 1). Petroselinic acid, the most active of the fatty acids examined, occurs naturally in several animals and plants such as *Apiaceae*, *Araliaceae*, *Griselinia*, *Garryaceae*, coriander (*Coriandrum sativum*), parsley (*Petroselinum crispum*), and carrots (*Daucus carota*) (16), and in the present study at low doses significantly inhibited biofilm formation by two MSSA and two MRSA strains without adversely affecting planktonic cell growth (Fig. 1 and 2). Furthermore, petroselinic acid suppressed the expressions of the virulence factors staphyloxanthin, lipase, and α-hemolysin by S. aureus (Fig. 4) and inhibited biofilm formation on nylon membranes and porcine skin without affecting cell morphology (Fig. 3 and 6). Most remarkably, petroselinic acid repressed the gene expressions of quorum sensing-related *agrA* and *RNAIII*, α-hemolysin (*hla*), and the virulence regulator *SaeR* (Fig. 5).

**FIG 7:**
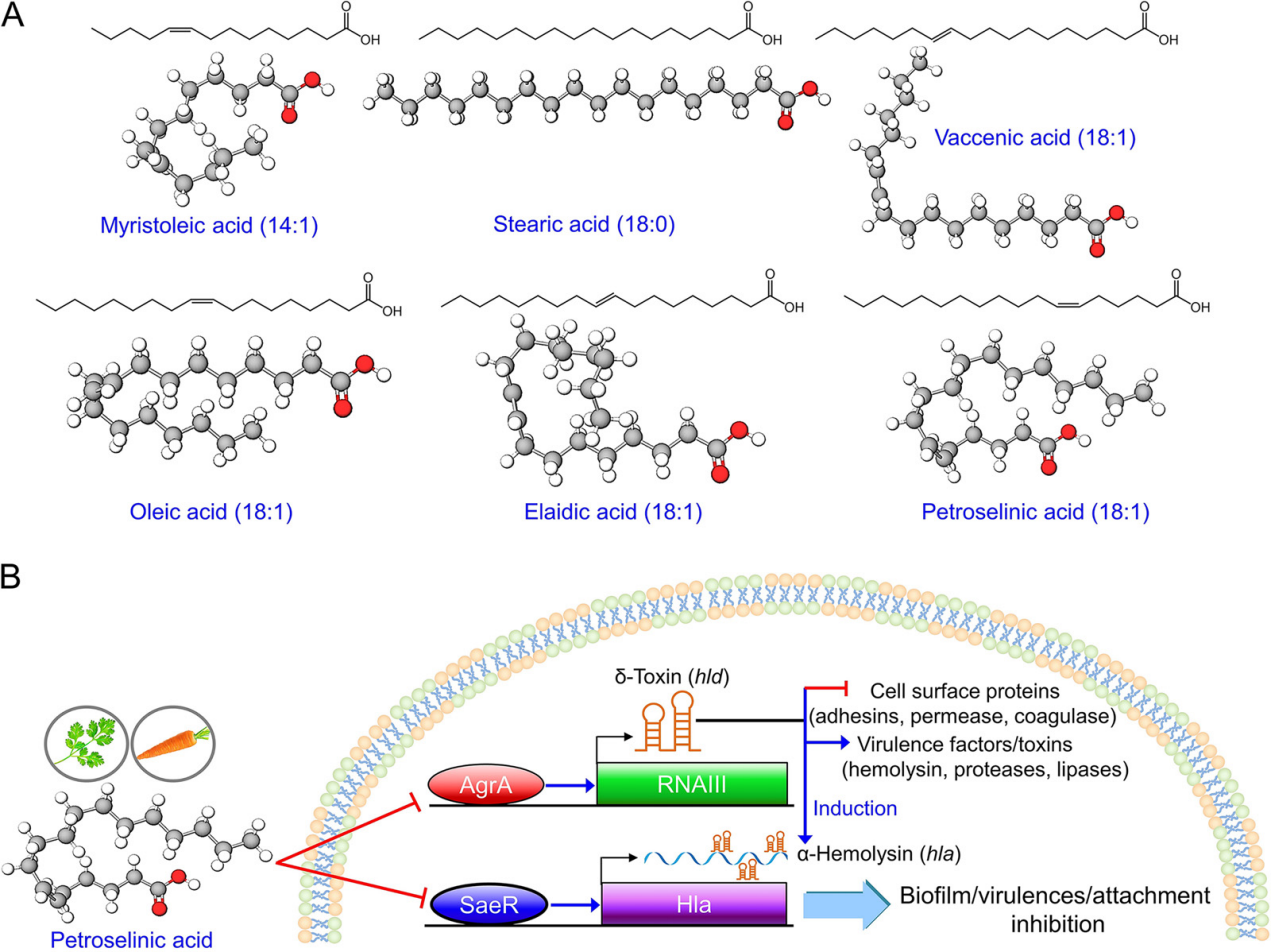
Ball and stick models of fatty acids and a schematic of the suggested mode of action of petroselinic acid in S. aureus. Chemical structures of six fatty acids (A). Putative mechanism for the effects of petroselinic acid in S. aureus (B).

Several fatty acids have been previously reported to exhibit antibiofilm activity against S. aureus at low concentrations. Reported examples include oleic acid (18:1 ω-9, *cis*) at 100 μg/mL (10), linoleic acid (18:2 ω-6) at 20 μg/mL (17), *cis*-11-eicosenoic acid (20:1 ω-9) and *cis*-11,14-eicosadienoic acid (20:2 ω-9) at 10 μg/mL (11), docosahexaenoic acid (DHA; C22:6, ω -3), and eicosapentaenoic acid (EPA; C20:5, ω-3) at 20 μg/mL (12), and myristoleic acid (14:1, ω-5) at 2 μg/mL inhibited S. aureus biofilm formation (13). In the present study, three C18 unsaturated fatty acids, namely vaccenic (18:1 ω-7), oleic (18:1 ω-9, *cis*), and petroselinic (18:1 ω-12) acids at 10–100 μg/mL, significantly inhibited S. aureus biofilm formation (Table 1 and Fig. 1), whereas other C18 fatty acids, such as steric (18:0) and elaidic (18:1 ω-9, *trans*) acids, failed to do so (Fig. 7A). These results suggest that carbon chain length, number of unsaturations, and positions and configurations of double bonds influence antibiofilm activity against S. aureus. Interestingly, previous transcriptomic results showed that docosahexaenoic and eicosapentaenoic acids inhibited the gene expressions of *RNAIII* and *hla* but not those of *agrA*, *nuc1*, and *saeR* (12), which is comparable to the current qRT-PCR results.

The observed downregulation of the *agrA*, *hla*, *nuc1*, *nuc2*, *RNAIII*, and *saeR* genes by petroselinic acid provide clues of the mechanism involved (Fig. 5). Previous reports indicate biofilm formation by S. aureus involves quorum sensing, proteases, nucleases, surface proteins, and other global regulators (4, 18). The S. aureus quorum-sensing system is encoded by the accessory gene regulator (*agr*) locus, which modulates the expression of virulence factors (19). Also, the *agr* system contributes to S. aureus virulence via biofilm development (20, 21). RNAIII is a regulatory RNA molecule of the *agr* locus (22), which stimulates the expression of several virulence factors such as δ-hemolysin *hld*, α-hemolysin *hla*, lipase, and exoprotease (23, 24). The biosynthesis of staphyloxanthin regulated by *agr* has been reported to occur in a RNAIII-independent manner. (23). Also, the virulence regulator *saeR* was reported to directly affect *hla* transcription (25), which concurs in part with the current result (Fig. 5). Overall, it appears that petroselinic acid diminished the virulence traits via repressing *agrA* and *RNAIII* and also that *hla* repression is due to the downregulation of *RNAIII* and *saeR* (Fig. 7). Since the protein structures of AgrA (26) and RNAIII (27) were revealed, it would be interesting to perform molecular docking studies between these QS regulators and fatty acids to find possible targets.

α-Hemolysin (Hla) plays an important role in S. aureus biofilm formation (28–30), and positive correlations between biofilm formation and α-hemolysin levels have been reported in studies on alizarin (31), azithromycin (32), flavonoids (33), norlichexanthone (34), stilbenoids (35), omega fatty acids (12), and lauric acid and myristic acid (36). Hence, our findings provide confirmatory evidence that petroselinic acid inhibits S. aureus biofilm formation by repressing the expression of α-hemolysin (Fig. 4 and 5). In addition, S. aureus lipases were found to promote biofilm formation (37), which is also in line with our results (Fig. 4).

The *agr* system contributes to S. aureus biofilm development (20) and biofilm dispersal (18). However, our results show that petroselinic acid inhibits S. aureus biofilm formation and represses the *agrA* and *RNAIII* system (Fig. 5), and petroselinic acid up to 500 μg/mL could not disperse established S. aureus ATCC 6538 biofilms (data not shown). In addition, it has been reported that extracellular DNA promoted S. aureus biofilm formation (4) and that extracellular nucleases perturb S. aureus biofilm formation (38). Our qRT-PCR results showed that petroselinic acid repressed the expressions of *nuc1* and *nuc2* (nuclease genes) in S. aureus (Fig. 5). These results indicate that biofilm inhibition by petroselinic acid is less associated with the *agr* dispersal system and nuclease activities.

The antioxidant, anti-inflammatory, and antiaging properties of petroselinic acid suggest its use in skin cosmetics (39). It was reported that petroselinic acid up to 1,000 μg/mL was not toxic to human peripheral blood mononuclear cells in an MTT assay (40). The results of the present study indicate that its potential use could be expanded to topical treatments for S. aureus infections, including MRSA infections, and the surface treatment of foods in processing facilities.

## MATERIALS AND METHODS

### Ethics statement.

For hemolysis assays, blood donors provided written consent before blood collection. The work was followed the instructions distributed by the Ethical Committee of Yeungnam University (Gyeongsan, South Korea).

### Bacterial strains, media, materials, and the growth analysis.

Two methicillin-sensitive S. aureus strains (MSSA; ATCC 6538, and ATCC 25923) and two methicillin-resistant S. aureus strains (MRSA; MW2, and ATCC 33591) were used in this work. Experiments on ATCC 6538 and ATCC 25923 strains were performed in Luria-Bertani (LB) broth, and those on ATCC 33591 and MW2 strains in LB liquid broth containing 0.2% glucose (all at 37°C). The 20 seven fatty acids, namely, butanoic acid (C4:0), pentanoic acid (C5:0), hexanoic acid (C6:0), heptanoic acid (C7:0), octanoic acid (C8:0), nonanoic acid (C9:0), decanoic acid (C10:0), undecanoic acid (C11:0), lauric acid (C12:0), myristic acid (C14:0), myristoleic acid (C14:1 ω-5), palmitic acid (C16:0), palmitoleic acid (16:1 ω-7), heptadecanoic acid (C17:0), stearic acid (C18:0), vaccenic acid (18:1 ω-7), oleic acid (18:1 ω-9, *cis*), elaidic acid (18:1 ω-9, *trans*), petroselinic acid (18:1 ω-12), conjugated linoleic acid (18:2 ω-6), α-linolenic acid (18:3 ω-3), γ-linolenic acid (18:3 ω-6), arachidonic acid (20:4 ω-6), behenic acid (22:0), erucic acid (22:1 ω-9), tricosanoic acid (C23:0), and nervonic acid (C24:1 ω-9) (Table 1) were purchased from MilliporeSigma (Burlington, MA, USA), Cayman Chemicals (Ann Arbor, MI, USA), or TCI Co. (Tokyo, Japan).

For free-floating cell growth measurements, optical densities were measured at 600 nm (OD_600_) using a spectrophotometer (Optizen 2120UV, Mecasys, Daejeon, Korea). MICs were determined by incubating cells in LB broth for 24 h in the presence of each fatty acid. To determine the cell growth for determining MIC, freshly grown colonies on agar plates were inoculated into LB broth and incubated for 16 h at 37°C with 250 rpm. After incubation, cells were diluted at 1:100 in fresh medium, added into each well of 96-well polystyrene plates (SPL Life Sciences, Pocheon, Korea), then cultivated for 24 h in the presence of each fatty acid. To determine the cell growth for determining MIC, optical densities were measured at 620 nm (OD_620_) using a plate reader, Multiskan SkyHigh Photometer (Thermo Fisher Scientific, Waltham, MA, USA).

### Crystal-violet biofilm assay.

The four S. aureus strains were conducted to a static biofilm formation assay by crystal violet staining on 96-well plates as previously described (41). Briefly, cells were inoculated into fresh LB broth at an initial turbidity of 0.05 at 600 nm (40 ± 5 × 10^6^ CFU/mL), and fatty acids (dissolved in DMSO) were added into each well of 96-well plates at various concentrations and cultivated for 24 h without agitation at 37°C. To measure biofilm formation, biofilms and free-floating cells were discarded and washed three times with water, and biofilm cells were stained with 300 μL of 0.1% crystal violet (dissolved in water) for 20 min. Crystal violet stained biofilm cells were then extracted by 300 μL of 95% ethanol with vigorous shaking for 90 sec. Absorbances were measured at 570 nm (OD_570_) using a Multiskan SkyHigh Photometer (Thermo Fisher Scientific). For biofilm dispersion assays, S. aureus was cultured in 96-well plates for 8 h without shaking at 37°C, and then petroselinic acid at various concentrations (50, 100, 200, and 400 μL/mL) was added and incubated for another 16 h. Biofilm formation results are presented as the averages of three independent cultures of 12 replicate wells.

### Microscopic observation of S. aureus biofilm cells.

Biofilm formation by S. aureus ATCC 6538 in 96-well plates (without agitation) in the presence or absence of petroselinic acid (0, 10, 20, 50, or 100 μg/mL) was investigated by live imaging microscopy using the iRiS Digital Cell Imaging System (Logos Biosystems, Anyang, Korea) and confocal laser scanning microscopy (CLSM, Nikon Eclipse Ti, Tokyo, Japan). After incubation for biofilm formation in 96-well plates for 24 h without agitation at 37°C, free-floating cells were disposed by washing three times with water, and biofilms were analyzed at different magnifications. Biofilm images were generated as color-coded 2D and 3D images using ImageJ (https://imagej.nih.gov/ij/index.html).

For confocal scanning microscopic (CLSM) analysis, only biofilm cells were stained with carboxyfluorescein diacetate succinimidyl ester (CFSE; Invitrogen, Molecular Probes Inc., Eugene, OR, USA) for 20 min, which stains viable cells in biofilms after discarding free-floating cells by washing with water, as previously reported (31). Stained S. aureus biofilms were visualized by CLSM using an Ar laser (excitation wavelength at 488 nm, and emission wavelength at 500 to 550 nm) and a 20× objective. Color confocal images were generated using software of NIS-Elements C version 3.2 total imaging solution (Nikon eclipse). For each sample, at least 12 random positions in two independent cultures were observed per experiment, and 20 planar images were analyzed per position. To quantify and measure biofilm formation, COMSTAT biofilm software (42) was used to investigate biovolumes (μm^3^/μm^2^), mean thicknesses (μm), and substratum coverages (%). Same thresholds were chosen for all image stacks, and at least 4 positions and 20 planar images were analyzed per sample.

SEM was also conducted to evaluate biofilm inhibition by petroselinic acid, as previously described (43). Briefly, 10 μL of overnight cultured S. aureus ATCC 6538 cells with or without petroselinic acid (0, 10, 20, 50, or 100 μg/mL) were inoculated into fresh 1 mL of LB (40 ± 5 × 10^6^ CFU/mL) in 96-well plates, and then a piece of sterile nylon filter membrane (0.4 × 0.4 mm square) was placed in each well carefully. Cells were then incubated without agitation for 24 h at 37°C. Biofilms on membranes were then fixed with a 2.5% glutaraldehyde/2% formaldehyde mixture for 24 h, post-fixed with OsO_4_ solution, and dehydrated using an ethanol series and 99% isoamyl acetate. After critical-point drying (HCP-2, Hitachi, Tokyo, Japan), biofilm cells on filters were sputter-coated with platinum and observed under an S-4800 scanning electron microscope (Hitachi, Tokyo, Japan) at magnifications of 3,000 to 20,000× and an accelerating voltage of 15 kV.

### Staphyloxanthin production assay.

The bright golden color of staphyloxanthin provides assay by optical observation (44, 45). Briefly, 20 μL of overnight cultured S. aureus ATCC 6538 cells were inoculated into 2 mL of fresh LB medium (40 ± 5 × 10^6^ CFU/mL) and incubated for 24 h with petroselinic acid (0, 10, 20, 50, or 100 μg/mL) at 37°C in 14-mL tubes with shaking at 250 rpm. Cells were then collected by centrifugation at 8,000 × *g* for 10 min, washed with sterile phosphate-buffered saline (PBS), resuspended in absolute ethanol (to extract the staphyloxanthin), and incubated at 40°C for 20 min. Cells were discarded by centrifugation at 10,000 × *g* for 10 min, and supernatant absorbances were measured at 450 nm (OD_450_).

### Lipase production assay.

To investigate the effect of petroselinic acid on extracellular lipase production by S. aureus, 20 μL of overnight cultured S. aureus ATCC 6538 cells were inoculated into 2 mL of LB (40 ± 5 × 10^6^ CFU/mL) and incubated with shaking at under 250 rpm for 20 h at 37°C with or without petroselinic acid (0, 10, 20, 50, or 100 μg/mL). Supernatants were then collected by centrifugation at 8,000 × *g* for 10 min, and 0.1 mL aliquots were mixed with 0.9 mL of substrate buffer (10% [vol/vol] of buffer A having 3 mg/mL of *p*-nitrophenyl palmitate in isopropyl alcohol and 90% [vol/vol] of buffer B having 1 mg/mL of gum arabic and 2 mg/mL sodium deoxycholate in Na_2_PO_4_ buffer [50 mM, pH 8.0]) and heated at 40°C in the dark for 30 min. Lipase reactions were then stopped by adding 1 M Na_2_CO_3_, and mixtures were centrifuged at 10,000 × *g* for 10 min. Supernatant absorbances were measured at 405 nm (OD_405_).

### Hemolysis assay.

The hemolysis of human red blood cells was investigated as described previously (41). Briefly, 20 μL of overnight cultured S. aureus MSSA 6538 cells were diluted in 2 mL of LB broth, incubated with or without petroselinic acid (0, 10, 20, 50, or 100 μg/mL) for 24 h with shaking at 250 rpm. Separately, human blood was centrifuged at 1,000 × *g* for 2 min, and cells were washed three times with PBS and dissolved gently in PBS at 3.3% (vol/vol). S. aureus culture (300 μL) was then added to 1 mL aliquots and incubated at 250 rpm shaking for 4 h at 37°C. Cells were removed by centrifuging at 16,600 × *g* for 10 min, and supernatants were collected and used to measure optical densities at 543 nm.

### RNA isolation and qRT-PCR.

S. aureus MSSA 6538 cells were inoculated into 15 mL of LB broth at 37°C in 250-mL flat bottom flasks at OD_600_ of 0.05 and then incubated for 6 h at 250 rpm shaking in the presence or absence of petroselinic acid (100 μg/mL). After incubation, RNase inhibitor (RNAlater, Ambion, TX, USA) was added before harvesting cells, and cells were immediately chilled in a dry ice bath containing 95% ethanol for 30 sec to prevent RNA degradation. To harvest cells, culture was spun down by centrifugation at 16,600 × *g* for 1 min, and total RNA was then isolated and purified using an RNA isolation/purification kit (Qiagen RNeasy minikit, Valencia, CA, USA).

qRT-PCR with gene specific primers was used to determine the transcriptional levels of 16 biofilm-related genes (*agrA*, *arlR*, *arlS*, *aur*, *icaA*, *hla*, *nuc1*, *nuc2*, *rbf*, *RNAIII*, *saeR*, *sarA*, *sarZ*, *seb*, *sigB*, and *spa*) in S. aureus MSSA 6538 cells. As the housekeeping control, 16s rRNA was used (Table S1 in the supplemental material). The qRT-PCR analysis technique was as previously described with some modification (31). qRT-PCR was assessed using a SYBR Green PCR master mix (Thermo Fisher Scientific, Waltham, MA) and an ABI StepOne real-time PCR system (Applied Biosystems, Foster City, USA). Gene expression levels were investigated by two independent experiments, and six reactions per gene were analyzed.

### Biofilm inhibition analysis on porcine skin.

The assay used was as previously described with some modification (46). Briefly, fresh quick-frozen porcine skin was obtained from the Korean Federation of Livestock Cooperatives (Seoul) and stored at −80°C until required. Skin pieces (0.5 × 0.5 cm) were sterilized before use by sequential immersion in 70% ethanol and 10% bleach solution for 20 min each. Skin pieces were then washed with sterile water 3 times. S. aureus MSSA 6538 cells in LB were added to the wells of a 12-well plate containing skin pieces and incubated with or without petroselinic acid (0, 10, 20, 50, or 100 μg/mL) for 24 h at 37°C without agitation. SEM was used to observe biofilm formation on skin pieces, as described above.

### Statistical analysis.

Data were evaluated by one-way ANOVA followed by Dunnett's test in SPSS version 23 (SPSS Inc., Chicago, IL, USA). Results are indicated as averages ± standard deviations, and statistical significance was acquired for *P* values < 0.05.

## References

[1] Dinges MM, Orwin PM, Schlievert PM. 2000. Exotoxins of *Staphylococcus aureus*. Clin Microbiol Rev 13:16–34. doi:10.1128/CMR.13.1.16. 10627489 PMC88931

[2] Stewart PS, Costerton JW. 2001. Antibiotic resistance of bacteria in biofilms. Lancet 358:135–138. doi:10.1016/s0140-6736(01)05321-1. 11463434

[3] Singhal D, Foreman A, Jervis-Bardy J, Bardy J-J, Wormald P-J. 2011. *Staphylococcus aureus* biofilms: nemesis of endoscopic sinus surgery. Laryngoscope 121:1578–1583. doi:10.1002/lary.21805. 21647904

[4] Arciola CR, Campoccia D, Speziale P, Montanaro L, Costerton JW. 2012. Biofilm formation in *Staphylococcus* implant infections. a review of molecular mechanisms and implications for biofilm-resistant materials. Biomaterials 33:5967–5982. doi:10.1016/j.biomaterials.2012.05.031. 22695065

[5] Rasko DA, Sperandio V. 2010. Anti-virulence strategies to combat bacteria-mediated disease. Nat Rev Drug Discov 9:117–128. doi:10.1038/nrd3013. 20081869

[6] Dickey SW, Cheung GYC, Otto M. 2017. Different drugs for bad bugs: antivirulence strategies in the age of antibiotic resistance. Nat Rev Drug Discov 16:457–471. doi:10.1038/nrd.2017.23. 28337021 PMC11849574

[7] Desbois AP, Smith VJ. 2010. Antibacterial free fatty acids: activities, mechanisms of action and biotechnological potential. Appl Microbiol Biotechnol 85:1629–1642. doi:10.1007/s00253-009-2355-3. 19956944

[8] Yoon BK, Jackman JA, Valle-Gonzalez ER, Cho NJ. 2018. Antibacterial free fatty acids and monoglycerides: biological activities, experimental testing, and therapeutic applications. Int J Mol Sci 19:1114. doi:10.3390/ijms19041114. 29642500 PMC5979495

[9] Kumar P, Lee J-H, Beyenal H, Lee J. 2020. Fatty acids as antibiofilm and antivirulence agents. Trends Microbiol 28:753–768. doi:10.1016/j.tim.2020.03.014. 32359781

[10] Stenz L, Francois P, Fischer A, Huyghe A, Tangomo M, Hernandez D, Cassat J, Linder P, Schrenzel J. 2008. Impact of oleic acid (*cis*-9-octadecenoic acid) on bacterial viability and biofilm production in *Staphylococcus aureus*. FEMS Microbiol Lett 287:149–155. doi:10.1111/j.1574-6968.2008.01316.x. 18754790

[11] Lee J-H, Kim Y-G, Park JG, Lee J. 2017. Supercritical fluid extracts of *Moringa oleifera* and their unsaturated fatty acid components inhibit biofilm formation by *Staphylococcus aureus*. Food Control 80:74–82. doi:10.1016/j.foodcont.2017.04.035.

[12] Kim Y-G, Lee J-H, Raorane CJ, Oh ST, Park JG, Lee J. 2018. Herring oil and omega fatty acids inhibit *Staphylococcus aureus* biofilm formation and virulence. Front Microbiol 9:1241. doi:10.3389/fmicb.2018.01241. 29963020 PMC6014104

[13] Kim Y-G, Lee J-H, Lee J. 2021. Antibiofilm activities of fatty acids including myristoleic acid against *Cutibacterium acnes* via reduced cell hydrophobicity. Phytomedicine 91:153710. doi:10.1016/j.phymed.2021.153710. 34461422

[14] Kim HS, Ham SY, Jang Y, Sun PF, Park JH, Lee JH, Park HD. 2019. Linoleic acid, a plant fatty acid, controls membrane biofouling via inhibition of biofilm formation. Fuel 253:754–761. doi:10.1016/j.fuel.2019.05.064.

[15] Grumezescu AM, Saviuc C, Chifiriuc MC, Hristu R, Mihaiescu DE, Balaure P, Stanciu G, Lazar V. 2011. Inhibitory activity of Fe_3_O_4_/oleic acid/usnic acid-core/shell/extra-shell nanofluid on *S. aureus* biofilm development. IEEE Trans Nanobioscience 10:269–274. doi:10.1109/TNB.2011.2178263. 22157076

[16] Ngo-Duy CC, Destaillats F, Keskitalo M, Arul J, Angers P. 2009. Triacylglycerols of Apiaceae seed oils: composition and regiodistribution of fatty acids. Eur J Lipid Sci Technol 111:164–169. doi:10.1002/ejlt.200800178.

[17] Kim Y-G, Lee J-H, Park JG, Lee J. 2020. Inhibition of *Candida albicans* and *Staphylococcus aureus* biofilms by centipede oil and linoleic acid. Biofouling 36:126–137. doi:10.1080/08927014.2020.1730333. 32093497

[18] Boles BR, Horswill AR. 2011. Staphylococcal biofilm disassembly. Trends Microbiol 19:449–455. doi:10.1016/j.tim.2011.06.004. 21784640 PMC3164736

[19] Ji G, Beavis R, Novick RP. 1997. Bacterial interference caused by autoinducing peptide variants. Science 276:2027–2030. doi:10.1126/science.276.5321.2027. 9197262

[20] Yarwood JM, Bartels DJ, Volper EM, Greenberg EP. 2004. Quorum sensing in *Staphylococcus aureus* biofilms. J Bacteriol 186:1838–1850. doi:10.1128/JB.186.6.1838-1850.2004. 14996815 PMC355980

[21] Boles BR, Horswill AR. 2008. *Agr*-mediated dispersal of *Staphylococcus aureus* biofilms. PLoS Pathog 4:e1000052. doi:10.1371/journal.ppat.1000052. 18437240 PMC2329812

[22] Koenig RL, Ray JL, Maleki SJ, Smeltzer MS, Hurlburt BK. 2004. *Staphylococcus aureus* AgrA binding to the RNAIII-*agr* regulatory region. J Bacteriol 186:7549–7555. doi:10.1128/JB.186.22.7549-7555.2004. 15516566 PMC524880

[23] Queck SY, Jameson-Lee M, Villaruz AE, Bach TH, Khan BA, Sturdevant DE, Ricklefs SM, Li M, Otto M. 2008. RNAIII-independent target gene control by the *agr* quorum-sensing system: insight into the evolution of virulence regulation in *Staphylococcus aureus*. Mol Cell 32:150–158. doi:10.1016/j.molcel.2008.08.005. 18851841 PMC2575650

[24] Chu AJ, Qiu YY, Harper R, Lin L, Ma C, Yang X. 2020. Nusbiarylins inhibit transcription and target virulence factors in bacterial pathogen *Staphylococcus aureus*. Int J Mol Sci 21:5772. doi:10.3390/ijms21165772. 32796751 PMC7461214

[25] Gudeta DD, Lei MG, Lee CY. 2019. Contribution of *hla* regulation by SaeR to *Staphylococcus aureus* USA300 pathogenesis. Infect Immun 87:e00231-19. doi:10.1128/IAI.00231-19. 31209148 PMC6704604

[26] Sidote DJ, Barbieri CM, Wu T, Stock AM. 2008. Structure of the *Staphylococcus aureus* AgrA LytTR domain bound to DNA reveals a beta fold with an unusual mode of binding. Structure 16:727–735. doi:10.1016/j.str.2008.02.011. 18462677 PMC2430735

[27] Benito Y, Kolb FA, Romby P, Lina G, Etienne J, Vandenesch F. 2000. Probing the structure of RNAIII, the *Staphylococcus aureus agr* regulatory RNA, and identification of the RNA domain involved in repression of protein A expression. RNA 6:668–679. doi:10.1017/s1355838200992550. 10836788 PMC1369947

[28] Anderson MJ, Lin YC, Gillman AN, Parks PJ, Schlievert PM, Peterson ML. 2012. Alpha-toxin promotes *Staphylococcus aureus* mucosal biofilm formation. Front Cell Infect Microbiol 2:64. doi:10.3389/fcimb.2012.00064. 22919655 PMC3417397

[29] Caiazza NC, O'Toole GA. 2003. Alpha-toxin is required for biofilm formation by *Staphylococcus aureus*. J Bacteriol 185:3214–3217. doi:10.1128/JB.185.10.3214-3217.2003. 12730182 PMC154062

[30] Scherr TD, Hanke ML, Huang O, James DB, Horswill AR, Bayles KW, Fey PD, Torres VJ, Kielian T. 2015. *Staphylococcus aureus* biofilms induce macrophage dysfunction through leukocidin AB and alpha-toxin. mBio 6:e01021-15. doi:10.1128/mBio.01021-15. 26307164 PMC4550693

[31] Lee J-H, Kim Y-G, Ryu SY, Lee J. 2016. Calcium-chelating alizarin and other anthraquinones inhibit biofilm formation and the hemolytic activity of *Staphylococcus aureus*. Sci Rep 6:19267. doi:10.1038/srep19267. 26763935 PMC4725881

[32] Gui Z, Wang H, Ding T, Zhu W, Zhuang X, Chu W. 2014. Azithromycin reduces the production of alpha-hemolysin and biofilm formation in *Staphylococcus aureus*. Indian J Microbiol 54:114–117. doi:10.1007/s12088-013-0438-4. 24426177 PMC3889843

[33] Cho HS, Lee JH, Cho MH, Lee J. 2015. Red wines and flavonoids diminish *Staphylococcus aureus* virulence with anti-biofilm and anti-hemolytic activities. Biofouling 31:1–11. doi:10.1080/08927014.2014.991319. 25535776

[34] Baldry M, Nielsen A, Bojer MS, Zhao Y, Friberg C, Ifrah D, Glasser Heede N, Larsen TO, Frøkiær H, Frees D, Zhang L, Dai H, Ingmer H. 2016. Norlichexanthone reduces virulence gene expression and biofilm formation in *Staphylococcus aureus*. PLoS One 11:e0168305. doi:10.1371/journal.pone.0168305. 28005941 PMC5179057

[35] Lee K, Lee J-H, Ryu SY, Cho MH, Lee J. 2014. Stilbenes reduce *Staphylococcus aureus* hemolysis, biofilm formation, and virulence. Foodborne Pathog Dis 11:710–717. doi:10.1089/fpd.2014.1758. 25007234

[36] Kim Y-G, Lee J-H, Park S, Kim S, Lee J. 2022. Inhibition of polymicrobial biofilm formation by saw palmetto oil, lauric acid and myristic acid. Microb Biotechnol 15:590–602. doi:10.1111/1751-7915.13864. 34156757 PMC8867970

[37] Nguyen MT, Luqman A, Bitschar K, Hertlein T, Dick J, Ohlsen K, Broker B, Schittek B, Gotz F. 2018. Staphylococcal (phospho)lipases promote biofilm formation and host cell invasion. Int J Med Microbiol 308:653–663. doi:10.1016/j.ijmm.2017.11.013. 29203111

[38] Beenken KE, Spencer H, Griffin LM, Smeltzer MS. 2012. Impact of extracellular nuclease production on the biofilm phenotype of *Staphylococcus aureus* under *in vitro* and *in vivo* conditions. Infect Immun 80:1634–1638. doi:10.1128/IAI.06134-11. 22354028 PMC3347440

[39] Alaluf SSB, Hu H-L, Green RMSB, Powell RJSB, Rawlings VASB, Rogers SJSB, Watkinson ASB, Cain WF. February 2010. Cosmetic use of petroselinic acid. European patent DE69927466T3.

[40] Ramanathan S, Ravindran D, Arunachalam K, Arumugam VR. 2018. Inhibition of quorum sensing-dependent biofilm and virulence genes expression in environmental pathogen *Serratia marcescens* by petroselinic acid. Antonie Van Leeuwenhoek 111:501–515. doi:10.1007/s10482-017-0971-y. 29101490

[41] Kim Y-G, Lee J-H, Kim S-I, Baek K-H, Lee J. 2015. Cinnamon bark oil and its components inhibit biofilm formation and toxin production. Int J Food Microbiol 195:30–39. doi:10.1016/j.ijfoodmicro.2014.11.028. 25500277

[42] Heydorn A, Nielsen AT, Hentzer M, Sternberg C, Givskov M, Ersboll BK, Molin S. 2000. Quantification of biofilm structures by the novel computer program COMSTAT. Microbiology 146:2395–2407. doi:10.1099/00221287-146-10-2395. 11021916

[43] Lee J-H, Kim Y-G, Khadke SK, Yamano A, Watanabe A, Lee J. 2019. Inhibition of biofilm formation by *Candida albicans* and polymicrobial microorganisms by nepodin via hyphal-growth suppression. ACS Infect Dis 5:1177–1187. doi:10.1021/acsinfecdis.9b00033. 31055910

[44] Lee J-H, Cho HS, Kim Y, Kim J-A, Banskota S, Cho MH, Lee J. 2013. Indole and 7-benzyloxyindole attenuate the virulence of *Staphylococcus aureus*. Appl Microbiol Biotechnol 97:4543–4552. doi:10.1007/s00253-012-4674-z. 23318836

[45] Daum RS. 2008. Removing the golden coat of *Staphylococcus aureus*. N Engl J Med 359:85–87. doi:10.1056/NEJMcibr0803278. 18596277

[46] Yang QP, Phillips PL, Sampson EM, Progulske-Fox A, Jin SG, Antonelli P, Schultz GS. 2013. Development of a novel ex vivo porcine skin explant model for the assessment of mature bacterial biofilms. Wound Repair Regen 21:704–714. doi:10.1111/wrr.12074. 23927831

