# Supplementary material for: Inhibition of Staphylococcus aureus Biofilm Formation and Virulence Factor Production by Petroselinic Acid and Other Unsaturated C18 Fatty Acids
Source: Microbiol Spectr. 2022 Jun 1;10(3):e01330-22. doi: 10.1128/spectrum.01330-22 (PMC9241682; doi:10.1128/spectrum.01330-22)
Supplement: Supplemental file 1 — Table S1. Download spectrum.01330-22-s0001.pdf, PDF file, 0.03 MB [file spectrum.01330-22-s0001.pdf]

Supplementary materials

Table S1. Sequences of the primers used for quantitative RT-PCR.

Gene	Name	Primer
agrA	Quorum-sensing regulator A	Forward 5'-TGA TAA TCC TTA TGA GGT GCT T-3'<br>Reverse 5'-CAC TGT GAC TCG TAA CGA AAA-3'
arlR	Response regulator	Forward 5'-TTA CGG TGC AGG CGA TTA TAT AG-3'<br>Forward 5'-TAC CGT TGA CAT CGA TAA TAT CC-3'
arlS	Histidine-protein kinase	Forward 5'-TGG AAT ACC AAT TCC ATG ATC T-3'<br>Forward 5'-TGC AAT CAA ATA TGA TGT GAA GAA-3'
aur	Zinc metalloproteinase aureolysin	Forward 5'-ACC GTG TGT TAA TTC GTG TGC TA-3'<br>Reverse 5'-ATG GTC GCA CAT TCA CAA GTT T-3'
icaA	Intercellular adhesion A	Forward 5’-TGA ACC GCT TGC CAT GTG-3'<br>Reverse 5’-CAC GCG TTG CTT CCA AAG A-3'
hla	α-Hemolysin	Forward 5'-CGG CAC ATT TGC ACC AAT AAG GC-3'<br>Reverse 5'-GGT TTA GCC TGG CCT TCA GC-3'
nuc1	Nuclease	Forward 5'-CAC CTG AAA CAA AGC ATC CTA A-3'<br>Reverse 5'-TAT ACG CTA AGC CAC GTC CAT-3'
nuc2	Nuclease	Forward 5'-ATG GAC GTG GCT TAG CGT AT-3'<br>Reverse 5'-TGA CCT GAA TCA GCG TTG TC-3'
rbf	Regulator of biofilm formation	Forward 5'-TTA GAA GGA ATC TTT AAA ACC TTA TTG AAT AA-3'<br>Reverse 5'-TTG TGA ATT TTT CTT CTT CGG ACA-3'
RNAIII	Transcriptional regulator	Forward 5'-ATC GAC ACA GTG AAC AAA TTC AC-3'<br>Forward 5'-CTC TAC TAG CAA ATG TTA CTC AC-3'
saeR	Response regulator	Forward 5'-GCC TTA ACT TTA GGT GCA GAT GAC TAT GTC-3'<br>Forward 5'-CGA CAG TTG TTC AAC TGG TTG ATG ATG G-3'
sarA	Transcriptional regulator	Forward 5'-GAG TTG TTA TCA ATG GTC-3'<br>Reverse 5'-GTT TGC TTC AGT GAT TCG-3'
sarZ	HTH-type transcriptional regulator	Forward 5'-CCT ATA CTG GTT ACA TTG TTT TAA TGG -3'<br>Forward 5'-TGG TGT CAG TGT TCC AGA ATC -3'
seb	Enterotoxin B	Forward 5'-TGT TCG GGT ATT TGA AGA TGG -3'<br>Reverse 5'-CGT TTC ATA AGG CGA GTT GTT-3'

sigB	RNA Polymerase sigma factor	Forward 5'-AAG TGA TTC GTA AGG ACG TCT-3'<br>Reverse 5'-TCG ATA ACT ATA ACC AAA GCC T-3'
spa	Protein A	Forward 5'-ACC AGA AAC TGG TGA AGA AAA TCC-3'<br>Reverse 5'-TAA CGC TGC ACC TAA GGC TAA TG-3'
16S rRNA	A component of ribosomes	Forward 5'-TGT TTG ACG ATG TTT GAG CA-3'<br>Reverse 5'-CCT TCC TCC AGT TCA GAT GC -3'
